# Nitroglycerin 0.4% ointment vs placebo in the treatment of pain resulting from chronic anal fissure: a randomized, double-blind, placebo-controlled study

**DOI:** 10.1186/1471-230X-13-106

**Published:** 2013-07-01

**Authors:** Scott M Berry, Charles F Barish, Raj Bhandari, Gemma Clark, Gregory V Collins, Julian Howell, John E Pappas, Dennis S Riff, Michael Safdi, Ann Yellowlees

**Affiliations:** 1 CRC of Jackson, 501 Marshall Street #500, Jackson, MS 39202, USA; 2 Wake Research Associates, LLC, 3100 Blue Ridge Road, Suite 300, Raleigh, NC 27612, USA; 3 Delta Research Partners, LLC, 608 Grammant Street, Monroe, LA 71201, USA; 4 ProStrakan Pharmaceuticals Ltd, Galabank Business Park, Galashiels TD1 1QH, UK; 5 Charlotte Clinical Research, 330 Billingsley Road, Charlotte, NC 28211, USA; 6 Kentucky Medical Research Center, 354 Waller Avenue, Suite 110, Lexington, KY 40504, USA; 7 Advanced Clinical Research Institute, 1211 West La Palma Ave. Suite 303, Anaheim, CA 92801, USA; 8 Consultants for Clinical Research/GCGA Physicians, 2925 Vernon Place, Suite 200, Cincinnati, OH, USA; 9 Quantics Consulting, Roslin BioCentre, Edinburgh, Scotland, UK

**Keywords:** Clinical Trial, Fissure In Ano, Nitroglycerin, Pain

## Abstract

**Background:**

Complications of chronic anal fissure (CAF) treatments are prompting interest in lower-risk therapies. This study was conducted to compare nitroglycerin (NTG) 0.4% ointment with placebo for pain associated with CAF.

**Methods:**

In this randomized, double-blind, placebo-controlled trial, patients with one CAF and moderate-to-severe pain (≥50 mm on a 100 mm visual analog scale [VAS]) received 375 mg NTG 0.4% (1.5 mg active ingredient) or 375 mg placebo ointment applied anally every 12 hours for 21 days. The primary end point was change from baseline VAS score in 24-hour pain averaged over days 14–18. Review of data from patients who withdrew early was blinded to treatment. To control for the confounding effects of analgesics, all patients received 650 mg acetaminophen for headache prophylaxis before each application.

**Results:**

A total of 247 patients were enrolled (NTG, n = 123; placebo, n = 124). The prespecified baseline observation carried forward (BOCF) analysis found no significant difference between groups; however, a last observation carried forward (LOCF) analysis showed a significant advantage for NTG. A post hoc analysis (LOCF/BOCF hybrid) demonstrated a significant adjusted mean difference of −7.0 mm in favor of NTG 0.4% (95% CI −13.6, –0.4; *P* = .038). Headache was the most common adverse event in the NTG (69.9%) and placebo (47.6%) groups.

**Conclusions:**

This was the first placebo-controlled study that also controlled for the confounding effects of analgesics used to treat NTG-induced headache. In patients with moderate-to-severe CAF pain, NTG 0.4% ointment effectively reduced CAF pain compared with placebo.

**Trial registration:**

ClinicalTrials.gov, NCT00522041

## Background

The first approved prescription product to treat chronic anal fissure (CAF) pain in the United States is nitroglycerin (NTG) 0.4% ointment (Rectiv^®^, ProStrakan, Inc., Bedminster NJ). Before introduction of this ointment, patients were limited to obtaining topical NTG from compounding pharmacies, risking nonstandard doses, contamination, and unknown homogeneity and stability. Data indicate that 46% of NTG ointments prepared by compounding pharmacies for the treatment of CAF do not meet US Pharmacopeia standards for potency and content uniformity [1].

Placebo-controlled trials investigated regimens of NTG 0.1%, 0.2%, and 0.4% ointment and determined NTG 0.4% twice daily to be the optimal dose for greatest analgesic effect compared with placebo in patients with CAF, without a significant increase in adverse events (AEs) over the other NTG concentrations [2]. However, few studies to date have specifically investigated the efficacy and safety of 0.4% NTG for the treatment of pain associated with CAF. The current study further examines the effects of NTG 0.4% ointment for moderate-to-severe pain associated with CAF. Dose strengths of NTG below 0.2% were not investigated here as prior controlled study evidence had indicated that 0.4% BID provided efficacy with an acceptable safety profile.

## Methods

### Study design and objective

This was a randomized, double-blind, placebo-controlled, multinational study to compare the effects of NTG 0.4% and placebo on pain associated with CAF. Pain relief was chosen as an end point because current data on the effects of NTG on healing rate are inconclusive, so the primary benefit to patients of using NTG is pain relief. The planned treatment duration was 21 days, as earlier studies with NTG had shown no increase in pain difference from placebo after 21 days. Specific design elements of the study were developed in discussion with the US Food and Drug Administration (FDA), including the primary and other end points, inclusion and exclusion criteria, vital signs and other clinical measurements, and inclusion of a standard oral analgesic regimen for both study arms to avoid any confounding effect of medications taken for headache.

The study conformed to the principles of the International Conference on Harmonisation guideline: Good Clinical Practice and the Declaration of Helsinki [3,4]. The study protocol, including changes, was reviewed and approved by institutional review boards and independent ethics committees, and all patients provided voluntary written informed consent. The trial is registered at ClinicalTrials.gov, no. NCT00522041. All sites were monitored, and all data were 100% verified against sources.

### Participants

Women and men aged 18 to 75 years were eligible to participate if they had a single, chronic posterior midline anal fissure accompanied by anal pain during the 6 weeks prior to screening and a 24-hour average pain score of ≥50 mm (on a 100-mm visual analog scale [VAS]) at baseline and on 2 of the 4 days before randomization, indicating moderate-to-severe pain. Evidence of at least one of the following criteria for chronic fissure also was required for study inclusion: sentinel skin tag, hypertrophied anal papillae, exposed internal anal sphincter, fibrotic fissure margins, or fibrotic anal sphincter. Patients had to be willing to discontinue or avoid nonprescription and prescription medicine (apart from conservative care fiber supplements, adequate fluid, and sitz baths) for the treatment of CAF for the duration of the study, including nonsteroidal anti-inflammatory drugs, acetylsalicylic acid (aspirin; except at low doses for cardiovascular prophylaxis [162 mg/day or ≤325 mg on alternate days]), acetaminophen (paracetamol), and any other analgesic for the treatment of headache or any other condition.

Patients were ineligible if they had more than one anal fissure, fistula-in-ano or anal abscess, inflammatory bowel disease, fibrotic anal stenosis, anal fissure secondary to an underlying condition, or a history of anal surgery. Additional key exclusion criteria included a recent history of migraine or chronic headaches; hypotension or uncorrected hypovolemia; increased intracranial pressure or inadequate cerebral circulation; use of nitroglycerin or any other nitric oxide donors, potassium channel blockers, calcium channel blockers, phosphodiesterase type 5 inhibitors, or any medications that could cause a drop in blood pressure when given in combination with nitrates; and the presence of any chronic pain requiring treatment with prohibited medications.

### Randomization and treatment

Eligible patients were randomized to receive NTG 0.4% ointment or placebo via an interactive voice response system using a randomization schedule prepared by an independent statistician. The randomization schedule was stratified by severity of pain as indicated by baseline VAS score (moderate 50–69 mm; severe ≥70 mm) and by gender. Gender was chosen as a stratification variable in case there were gender differences in response to NTG or placebo.

Patients in the NTG group applied NTG 375 mg 0.4% ointment (containing 1.5 mg active ingredient) twice daily. Patients in the placebo group applied placebo 375 mg ointment into the anal canal twice daily. The dose was measured by the patients using a measuring line, with the study medication being delivered from tubes. Compliance with treatment was determined by measuring the weight of the tubes. Placebo and NTG tubes were identical and could not be identified as NTG or placebo by either patient or physician. Placebo ointment was vehicle only and, apart from the lack of active ingredient, was identical to the NTG ointment. Thus, excipients and appearance/feel of the placebo ointment was the same as that of the active NTG. All patients were instructed to take acetaminophen 650 mg 30 minutes before each application to ensure consistent prophylaxis for headache (a known AE of NTG) and to reduce any confounding effect on reported pain, as previously described.

### Outcome measures

The primary end point was absolute change from baseline in patient-reported 24-hour average CAF pain intensity as measured using the 100-mm VAS over days 14 to 18 of treatment. The VAS has been shown to be a reliable measurement of acute pain [5] and is a validated tool to measure clinically important changes in pain severity [6]. The pain score was averaged over days 14 to 18 to minimize the effect of a bowel movement on pain scores on an individual day.

Secondary end points were as follows: time to improvement in 24-hour average VAS (defined as a decrease of 10 mm and 50% from baseline); patients’ global assessment of treatment at day 21 (last assessed visit day); percentage of responders, defined as patients with a decrease in 24-hour average pain intensity VAS score of ≥10 mm and ≥50% from baseline for treatment days 14 to 18; and absolute change in 24-hour average VAS score at days 7, 14, and 21 (not reported).

Five clinic visits were scheduled (screening and days 0 [randomization], 7, 14, and 21). Patient global assessment of 24-hour average pain intensity (VAS score) was obtained on day 0 and daily thereafter at bedtime until the evening before the day 21 assessment. Patients completed their final VAS and assessment of therapy on day 21 at the clinic. Patients who withdrew early from the study were asked to continue to record VAS scores until day 21.

Safety assessments included physical examination findings, vital signs (blood pressure and heart rate), orthostatic hypotension assessment, laboratory tests (hematology, serum chemistry, and urinalysis), 12-lead electrocardiograms (ECGs), and AEs and serious AEs.

Patients were instructed to complete daily diary cards to record time of administration of study medication (NTG ointment and acetaminophen), VAS assessments, number of sitz baths, and incidence and severity of headaches. For pain, patients were asked to record their response to the question “What has been your average pain today?”on a VAS at the end of each day.

### Statistical analysis

#### Primary end point

The intent-to-treat (ITT) population, comprising all randomized patients who received at least one dose of study drug, was the primary analysis population. The primary efficacy end point (absolute change from baseline in 24-hour average CAF pain intensity for days 14 to 18 of treatment) was assessed using an analysis of covariance (ANCOVA) model with treatment and gender as factors and baseline VAS pain score as a covariate. To compare treatment groups, all statistical tests were two-sided and performed using a 5% significance level, leading to 95% (two-sided) confidence intervals (CIs).

For the primary end point, different approaches were used to handle missing VAS data. For patients who withdrew before day 18, a zero change from baseline VAS score was imputed (ie, baseline observation carried forward [BOCF]). For patients who did not withdraw before day 18, any individual missing VAS scores on days 14 to 18 were calculated as the average of available scores between days 14 and 18. If all VAS scores were missing between days 14 and 18 but the patient had not withdrawn from the study before day 18, the last nonmissing value was carried forward (ie, last observation carried forward [LOCF]).

A sensitivity analysis was also planned to assess the effect of missing data if more than 5% of patients had missing data. This analysis used the average of available VAS scores for days 14 to 18 (if any were available) or the last nonmissing score up to day 18. If patients received any other treatment after day 14 (or if treatment was unknown), calculations were based on either (1) average VAS scores on days 14 to 18 prior to any other treatment or (2) the last nonmissing score up to day 18 if no scores existed for days 14 through 18. If patients received any treatment before day 14 (or treatment unknown), the last nonmissing VAS score prior to any treatment was used. This supportive LOCF analysis was prespecified to show what effect patient withdrawals may have had on the primary BOCF analysis. It was only triggered in the event of a significant number of withdrawals (5% of all cases), which was indeed the case. In addition, a supportive analysis on absolute change from baseline VAS score was performed on the ITT population using a repeated measures ANCOVA. All post-baseline VAS scores up to (but excluding) the assessment made at the last visit day (this assessment was not made in the evening so was not consistent with other VAS scores) were included until day 23. This model assumed that any missing data were missing at random and an autoregressive first order variance–covariance structure should be used.

Because of concerns about the BOCF approach being too conservative and the LOCF approach being too generous in their management of missing data, a third approach was recommended during regulatory review. In this LOCF/BOCF hybrid analysis, an independent Data Review Committee (DRC) previously unaffiliated with the current study analyzed blinded data (eg, pain scores, AE reports, study drug treatment duration, post-study treatments for anal fissure) from patients for evidence of early withdrawal due to pain relief. When evidence of early effective pain relief was found, actual recorded pain VAS scores from days 14 through 18 were used when available; when these scores were unavailable, scores obtained before day 14 were used (LOCF). For patients who withdrew without evidence of early pain relief, a zero change was imputed (BOCF).

#### Secondary end points

Secondary end points of time to improvement and percentage of responders were analyzed for the ITT population using a hierarchical test procedure in which sequential hypothesis testing was stopped when results failed to reject the null hypothesis of no difference in an end point at the 5% significance level. Analyses were performed in the following order: time to improvement for a 50% decrease in 24-hour average pain intensity (VAS); time to improvement for a 10-mm decrease in 24-hour average pain intensity (VAS); percentage of responders who had a 50% decrease in 24-hour average pain intensity from baseline to primary end point; and percentage of responders who had a 10-mm decrease in 24-hour average pain intensity (VAS) from baseline to primary end point. Absolute change in 24-hour average VAS at days 7, 14, and 21 was analyzed using the same ANCOVA model as the initial primary efficacy analysis. The log-rank test was used to compare time to improvement between groups stratified by baseline VAS and gender. Patients’ global assessment of therapy at day 21 was compared between treatment groups using logistic regression with treatment and gender as factors and baseline VAS pain as a covariate.

#### Safety

All AEs were analyzed according to severity and relationship to NTG 0.4%, with headache summarized separately and together with other AEs. Severity was rated by the investigator as mild (no limitation of usual activities), moderate (some limitation of usual activities), or severe (inability to undertake usual activities).

#### Sample size

The required sample size was determined to be 246 (123 per treatment group) to detect a difference in VAS score of 10 mm (equivalent to 20% for a patient with a baseline VAS of 50 mm), assuming a 24 mm standard deviation, with significance level of 5% and power 90% using a 2-sided test.

## Results

**Figure 1:**
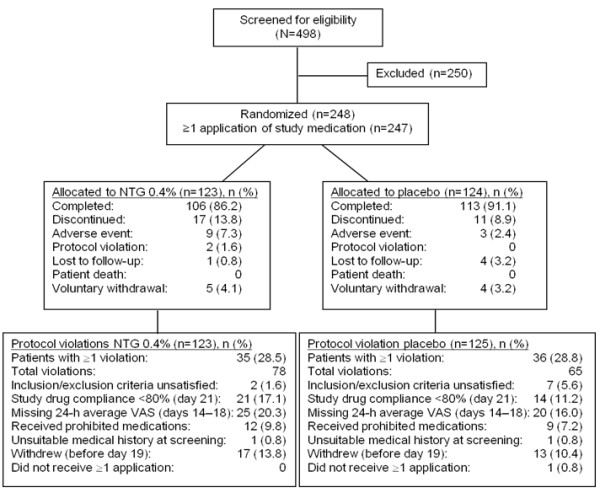
**Patient disposition.** NTG, nitroglycerin; VAS, visual analog scale.

The study took place at 45 sites in the United States, Argentina, Brazil, and Mexico between August 2007 and July 2008. The ITT population comprised 247 patients (NTG 0.4% n = 123; placebo n = 124). Patient disposition is shown in Figure 1.

### Participants

In the NTG and placebo groups, 106 of 123 (86.2%) and 113 of 124 (91.1%) patients, respectively, completed the study. There were 35 (28.5%) patients in the NTG group and 36 (28.8%) patients in the placebo group who had protocol violations, most of which were related to the timing of the administration of acetaminophen with respect to the application of NTG ointment. Median compliance with the application of ointment was 95% in both groups. However, 21 (17.1%) patients in the NTG group and 12 (9.7%) patients in the placebo group had <80% compliance. Median compliance with acetaminophen treatment was greater than 97% in both groups.

Patient demographics and baseline characteristics are shown in Table 1. The distribution of patients by age, gender, and race was similar between groups. The most common medical conditions reported for all 247 patients were hypertension (67 [27.1%] patients), constipation (27 [23.1%]), and hemorrhoids (50 [20.2%]). Patients were evenly distributed between groups with respect to baseline pain VAS score; approximately half of patients in each group had moderate baseline pain (VAS 50–69 mm), and the remainder had severe pain (VAS ≥70 mm).

**Table 1:** Patient demographics and baseline characteristics in intent-to-treat population

**Characteristic**	**NTG 0.4%**	**Placebo**	*P *value (2-sided)*
	**(n = 123)**	**(n = 124)**	
Mean age, y (SD)	46.5 (12.6)	43.4 (13.2)	.057
Male, n (%)	65 (52.8)	66 (53.2)	1
Race, n (%)			.640
White	99 (80.5)	96 (77.4)	
Black	21 (17.1)	16 (12.9)	
Baseline VAS, n (%)			.899
Moderate 50–69 mm	57 (46.3)	59 (47.2)	
Severe ≥70 mm	66 (53.7)	65 (52.0)	
Most common medical conditions, n (%) (≥20 patients in either treatment group)			
Gastrointestinal disorders			
Constipation	29 (23.6)	28 (22.6)	.881
Hemorrhoids	27 (22.0)	23 (18.5)	.530
Gastroesophageal reflux disease	20 (16.3)	17 (13.7)	.597
Other disorders			
Drug hypersensitivity	26 (21.1)	18 (14.5)	.187
Hypertension	38 (30.9)	29 (23.4)	.200
Hypercholesterolemia	23 (18.7)	15 (12.1)	.162

*NTG* nitroglycerin, *SD* standard deviation, *VAS* visual analog scale.

*Fisher’s exact test used for all between-group comparisons except age, which was compared using *t* test.

### Efficacy

#### Primary end point

For the primary end point of absolute change from baseline in 24-hour pain averaged over days 14 to 18, the adjusted mean (standard error [SE]) change from baseline VAS was −40.4 (3.1) mm in the NTG group and −34.9 (3.0) mm in the placebo group. The mean (95% CI) difference between groups was −5.4 (−12.3, 1.4) mm in favor of NTG, but this difference was not significant (*P* = .118).

The sensitivity analysis was performed because >5% of patients had missing primary end point data. This analysis showed a significant adjusted mean (95% CI) difference between groups in change from baseline in 24-hour average pain at days 14 to 18 in favor of NTG (−13.8, –0.6; *P* = .033).

**Figure 2:**
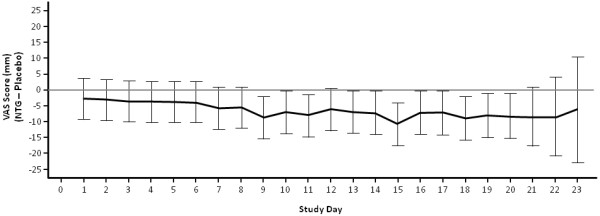
**Treatment difference in VAS pain at each time point: Adjusted Mean With 95% CI (ITT Population).** Includes all post-baseline VAS scores prior to withdrawal and up to (but excluding) the assessment made at the last visit day. Adjusted means and CIs were derived from a repeated measures ANCOVA model with an auto-regressive first-order structure, with treatment, time, country, gender, and treatment × time as factors and baseline VAS pain as a covariate. ANCOVA, analysis of covariance; ITT, intent-to-treat; NTG, nitroglycerin; VAS, visual analog scale.

The results of the supportive analysis on absolute change from baseline VAS score using a repeated measures ANCOVA model are shown in Figure 2. The adjusted mean change from baseline in VAS score was greater for the NTG group than for the placebo group at all time points. The mean (95% CI) treatment difference for the average of days 14 to 18 was −8.1 (−13.8, –2.3) mm in favor of NTG. The greatest adjusted mean difference between treatment groups occurred on day 15.

The results of the regulatory-specified analysis (LOCF/BOCF hybrid) are presented in Table 2. The estimated mean (95% CI) difference between treatment groups in the adjusted change from baseline VAS score was −7.0 (−3.6, –0.4) mm, a significant difference in favor of NTG (*P* = .038). Analysis by the DRC indicated that 9 of the 27 patients who withdrew were deemed to have done so because of early effective pain relief; 6 were in the NTG group and 3 were in the placebo group.

**Table 2:** Absolute change from baseline in 24-hour average pain response for days 14-18: LOCF/BOCF hybrid analysis

	**NTG 0.4%**	**Placebo**	**Difference**
	**(n = 123)**	**(n = 124)**	**NTG 0.4% – Placebo**
Day 0 (baseline)			
Mean (SD)	72.7 (14.5)	73.0 (13.2)	
Median (range)	73.0 (13, 100)	71.5 (51, 100)	
Days 14–18			
Mean (SD)	30.7 (26.6)	37.8 (27.8)	
Median (range)	20.6 (0, 94)	35.5 (0, 97)	
Change from baseline			
Mean (SD)	−42.0 (26.5)	−35.2 (27.4)	
Median	−45.2 (−91, 18)	−36.0 (−95, 12)	
Adjusted mean (SE)	−43.7 (3.0)	−36.7 (2.9)	−7.0 (3.3)
95% CI			−13.6, –0.4
*P* value			.038

*BOCF* baseline observation carried forward, *CI* confidence interval, *LOCF* last observation carried forward, *NTG* nitroglycerin, *SD* standard deviation, *SE* standard error.

#### Secondary end points

In the NTG group, 89 (72.4%) of 123 patients achieved a ≥50 decrease in VAS score compared with 80 of 124 (64.5%) patients in the placebo group. Mean time to improvement was 10.7 and 13.2 days, respectively (*P =* .071). The number of patients who had a ≥10 mm decrease in VAS score was 109 (88.6%) in the NTG group and 106 (85.5%) in the placebo group. Mean time to improvement was 6.1 and 7.2 days, respectively (*P =* .287).

In the analysis of patients’ global assessment of treatment at day 21, 95 (77.2%) patients in the NTG group and 102 (82.3%) patients in the placebo group answered that the benefit of their treatment outweighed any AE they experienced; this difference was not significant (*P* = .277). The difference between groups in the percentage of responders was also not statistically significant: 73 (59.3%) patients in the NTG group and 62 (50.0%) patients in the placebo group had a ≥50% decrease from baseline in 24-hour average pain intensity for days 14 to 18 (*P* = .131).

### Safety

The mean (SD) number of doses of study treatment was 36.4 (10.8) in the NTG group and 38.9 (8.7) in the placebo group. The most common AEs in the safety population, the severity of AEs, and the percentage of patients who had AEs leading to study discontinuation are shown in Table 3. A total of 96 (78.0%) of 123 patients in the NTG group and 67 (54.0%) of 124 patients in the placebo group experienced at least one AE during the study. The most common AEs overall were headache, dizziness, diarrhea, and nausea. The NTG group had a higher incidence of headache, but there were no other notable differences between groups. More patients in the NTG group (5.7%) than in the placebo group (0.8%) had an AE of headache that led to study discontinuation.

**Table 3:** Most common (≥2%) treatment-emergent adverse events by treatment group (safety population), severity, and study discontinuation

**TEAEs**	**NTG 0.4%**	**Placebo**
**n (%)**	**(n = 123)**	**(n = 124)**
Number of patients with ≥1 AE	96 (78.0)	67 (54.0)
Mild	31 (25.2)	15 (12.1)
Moderate	41 (33.3)	38 (30.6)
Severe	24 (19.5)	14 (11.3)
Headaches	86 (69.9)	59 (47.6)
Dizziness	6 (4.9)	2 (1.6)
Diarrhea	4 (3.3)	4 (3.2)
Nausea	2 (1.6)	5 (4.0)
Sinusitis	3 (2.4)	1 (0.8)
AEs leading to study discontinuation	9 (7.3)	4 (3.2)
Headache	7 (5.7)	1 (0.8)
Palpitations	1 (0.8)	0
Celiac disease	0	1 (0.8)
Diarrhea	0	1 (0.8)
Skin fissures	1 (0.8)	0
Pain of skin	0	1 (0.8)

Abbreviation: *AE* Adverse event, *TEAE* Treatment-emergent adverse event.

Of the 96 patients who reported AEs in the NTG group, 84 (68.3%) had AEs that were considered related to the study treatment. Of the 67 patients who reported AEs in the placebo group, 52 (41.9%) had AEs that were considered related to study treatment.

Severe AEs occurred in 24 (19.5%) patients in the NTG group and 14 (11.3%) in the placebo group. Three patients reported serious AEs, which were considered unrelated to study treatment: osteomyelitis (n = 1) and anal sphincterotomy (n = 1) in the NTG group and iron deficiency anemia (n = 1) in the placebo group. No deaths occurred during the study.

The most common AE in both groups was headaches, occurring in 86 (69.9%) of 123 patients in the NTG group and 59 (47.6%) of 124 patients in the placebo group. The severity and mean duration of headaches is shown in Table 4. Most headaches were mild or moderate in severity and decreased in frequency over time [7]; severe headaches were reported in 20 (16.3%) patients in the NTG group and 11 (8.9%) in the placebo group. The mean duration of headaches was 2.5 h in the NTG group and 4.4 h in the placebo group. Headache was the main AE that led to discontinuation in the NTG group (7 patients; Table 3). Dizziness, a possible symptom of orthostatic hypotension, was the second most common AE reported during the study. A trend toward decreased supine systolic and diastolic blood pressure was observed after application of NTG ointment; values remained steady after application of placebo ointment. Diastolic blood pressure was typically restored upon standing. Mild compensatory tachycardia was also evident in the NTG group, which is a normal physiologic compensation for decreased blood pressure. However, changes in blood pressure and heart rate in the NTG group at visit 1 were less evident at visit 2. No trends were noted in changes from baseline in hematology, serum chemistry, urinalysis, physical examination, or ECG. Anal fissures had resolved in 61 (49.6%) of 123 patients in the NTG group and 52 (41.9%) of 124 patients in the placebo group.

**Table 4:** Summary of headache adverse events (safety population)

**Headaches**	**NTG 0.4%**	**Placebo**
	**(n = 123)**	**(n = 124)**
Total number of occurrences	972	254
Mean (SD) duration, h	2.5 (3.5)	4.4 (6.4)
Patients with ≥1 headache, n (%)	86 (69.9)	59 (47.6)
Mild, n (%)	27 (22.0)	16 (12.9)
Moderate, n (%)	39 (31.7)	32 (25.8)
Severe, n (%)	20 (16.3)	11 (8.9)

Abbreviation: *NTG* Nitroglycerin, *SD* Standard deviation.

Hepatotoxicity markers, measured because of acetaminophen use in this study, were comparable between groups and presented no safety concerns. Most patients took at least one concomitant medication during the study: 99 (80.5%) of 123 patients in the NTG group and 90 (72.6%) of 124 patients in the placebo group. The most commonly reported medication was acetylsalicylic acid, taken by 16 (13.0%) patients in the NTG group and 14 (11.3%) patients in the placebo group.

## Discussion

In this patient population with moderate-to-severe CAF pain, the primary end point of absolute change in pain response was not significant between treatment groups in the prespecified analysis (BOCF) performed on this data set. However, a subsequent regulatory-requested post hoc analysis (LOCF/BOCF) found a statistically significant difference between treatment groups in favor of NTG (*P* = .038). Moreover, the sensitivity analysis, which included all reported changes in VAS scores for both treatment groups, also found a significant difference in efficacy between treatment groups in favor of NTG (*P* = .033).

Several issues in the conduct of the study and the prespecified analyses contributed to these results. Because more than 10% of all patients did not complete the study, the conservative BOCF method of imputing missing data for these patients may have been overly conservative if patients had a reduction in CAF pain and decided not to complete the study. This hypothesis is supported by the fact that the LOCF analysis showed a significant difference between the study groups. The sensitivity analysis took a less conservative approach and used the last available VAS scores at the time of discontinuation. As more patients in the NTG group discontinued early (13.8% vs 8.9% in the placebo group), the more conservative primary analysis led to a smaller, nonsignificant treatment difference than that determined in the sensitivity analysis. In addition, subsequent review of the 27 patients who withdrew determined that more patients in the NTG group who discontinued did so because of early effective pain relief (6 of 16 [37.5%] vs 3 of 11 [27.3%], respectively). The hybrid LOCF/BOCF approach was used a regulatory-requested analysis that also demonstrated a significant difference in the primary end point in favor of NTG.

Both groups demonstrated mean reductions in pain on VAS scores in the range of 30 to 40 mm and, in both groups, pain scores improved over time. Previous research supports a VAS score change in the range of 10 to 17 mm as clinically relevant in adults [6]. A placebo effect in CAF pain reduction is well documented and relates to any nonsurgical therapy [8-11]. However, time to improvement (≥50 decrease) in VAS scores was shorter in the NTG group compared with the placebo group, suggesting that patients receiving NTG experienced pain relief sooner than did patients in the placebo group. Moreover, the repeated measures analysis demonstrated that the adjusted mean change in VAS score from baseline was greater for the NTG group than for the placebo group at all time points.

Several prospective, randomized, controlled trials have been compared the efficacy and safety of topical NTG with other agents in the treatment of anal fissure, including lignocaine [12], botulinum toxin [13,14], and diltiazem [15]. Results varied across these studies, with NTG showing superior efficacy in 2 of the 4 trials [12,14]. However, the dosages of NTG used in these trials were lower than that used in the current trial, varying from 0.2% NTG applied twice daily [12,14,15] to 0.2% NTG three times daily [13], compared with 0.4% NTG twice daily in the current study.

The design of the current study (placebo-controlled, confirmed run-in period to measure the degree of pain, and confirmation of CAF) controlled for many of the known confounding factors of pain studies in CAF. However, it is possible that the prespecified and secondary efficacy end points in this study were confounded by the benefit from placebo ointment, investigator advice on diet and toileting, natural resolution of the anal fissure, or the twice-daily acetaminophen taken by all patients to minimize headache, which may not be representative of standard conservative care in the general population. However, it is likely that our measurements in the placebo group reflected a placebo ointment effect, rather than natural resolution of the fissure, as patients had CAF and moderate-to-severe pain for several days prior to randomization. This may be explained partially by the lubricating effect of the ointment. Furthermore, within the strict environment of this placebo-controlled trial, the absolute magnitude of difference between the NTG and placebo groups was low, but the magnitude of pain reduction from baseline in the NTG group was well within what is accepted as clinically meaningful. In addition, the dose of acetaminophen used was unlikely to have had a noticeable analgesic effect on moderate-to-severe pain from CAF. This is the first placebo-controlled study that has also controlled for the confounding effect of analgesics taken to treat NTG-induced headache.

Headache was the most common treatment-related AE. While more headaches occurred in the NTG group, they were of a shorter duration than those in the placebo group but more frequently reported to be moderate or severe. Although 69.9% of NTG patients reported headaches during the study, 77.2% of NTG patients stated that the benefit of the treatment they received outweighed any AEs. These data support previous reports on the use of NTG ointment for CAF, in which headaches were transient, also occurred in the placebo group, and did not interfere with treatment [16]. The incidence of headache in the placebo group (47.6%) may have been associated with the requirement for patients to complete a daily diary to record incidence and severity of headaches, potentially encouraging reporting of headaches. Specifically, patients were asked in the daily diary “Did you experience a headache today?” This approach may have inflated both the placebo and NTG headache responses over and above what is normally reported in other studies.

The protocol-specified use of acetaminophen to ensure consistent prophylaxis for headache could potentially have contributed to anal fissure pain relief (other NTG studies have not controlled for analgesic use). To control for the possible confounding factor of analgesic use, a prespecified dose of prophylactic acetaminophen was mandated in this study and all other analgesics prohibited. It is not proposed that patients should routinely receive acetaminophen as part of NTG therapy.

It is important to note that there was no attempt in the study protocol to confirm healing of the fissure or decrease the dose of NTG for patients who experienced severe headaches. These issues may be interesting topics for future studies.

## Conclusions

The common, painful condition of CAF would benefit from a clinically acceptable, low-risk, moderately priced, and reliably manufactured medication. The FDA recently approved an NTG 0.4% ointment, which appears to provide effective relief of CAF pain, without risk of sphincter injury and with an acceptable safety profile, and to offer a standardized formulation of an agent that had previously been given to patients through an unregulated pharmacy compounding process. In this randomized, double-blind, placebo-controlled trial, the first placebo-controlled trial that controlled for the confounding effect of analgesics taken to treat NTG-induced headache, NTG 0.4% effectively reduced moderate-to-severe CAF pain compared with placebo. The duration of treatment with NTG remains to be determined, but there is no evidence that continuing treatment beyond the resolution of pain symptoms is beneficial. In the longer term, the analgesic effect of NTG may allow spontaneous healing of CAF, avoiding the need for surgical intervention.

### Institutional review boards/ethics committees

*United States*—Central institutional review board (IRB) services were provided by: Schulmann Associates Institutional Review Board, Cincinnati, OH. Site-specific IRB responsibilities were carried out by Human Subjects Protection Office/Institutional Review Board, Penn State Milton S. Hershey Medical Center Hershey, PA; Scott & White Memorial Hospital Institutional Review Board, Temple, TX; William Beaumont Hospital Human Investigation Committee, Royal Oak, MI; and Institutional Review Board, Cleveland Clinic, Cleveland, OH.

*South America—*International ethics committees by investigative site were: Prof. Dr. Luis Maria Zieher Comité, Independiente de Ética para Ensayos en Farmacologia Clinica, Buenos Aires, Argentina; Comité de Docencia Sanantorio Amaricano S.A., Provincia de Santa Fe, Argentina; Comitë de Ética em Pesquisa em Seres Humanos do Hospital Geral de Goiânia Avenida anhanguera, Goiânia, GO, Brazil; Ministério da Saúde, Conselho Nacional de Saúde, Comissäo Nacional de Ética em Pesquisa, Esplanada dos Ministérios, Brazil.

*Mexico—*International ethics committees by investigative site were: Comite de Etica e Investigacion de Torre Medica del Pacifico, Acapulco, Guerrero CP; Comite de Etica y Enseñanza, Monterrey, Nuevo Leon CP; Comision de Etica e Investigacion del Hospital General, Durango, Durango, CP; Comite de Etica del Hospital General Regional de Leon, Leon, Guanajuato, CP; Comite de Etica del Hospital General Regional, Tuxtla Guitterrez, Chiapas. CP; Comité de Investigación y Etica CDI, Boca del rio, Veracruz, CP.

## Abbreviations

AE: Adverse events; ANCOVA: Analysis of covariance; BOCF: Baseline observation carried forward; CAF: Chronic anal fissure; CI: Confidence interval; DRC: Data Review Committee; ECG: Electrocardiogram; ITT: Intent to treat; LOCF: Last observation carried forward; NTG: Nitroglycerin; SD: Standard deviation; SE: Standard error; VAS: Visual analog scale.

## Competing interests

Authors’ declaration of personal interests. Scott Berry: Participation on Rectiv Advisory Board. Charles Barish: Nothing to disclose. Raj Bhandari: Nothing to disclose. Gemma Clark: Employee of ProStrakan Pharmaceuticals Ltd, Galabank Business Park, Galashiels, UK. Gregory Collins: Nothing to disclose. Julian Howell: Employee of ProStrakan Pharmaceuticals Ltd, Galabank Business Park, Galashiels, UK. John Pappas: Nothing to disclose. Dennis Riff: Nothing to disclose. Michael Safdi: Nothing to disclose. Ann Yellowlees: Employee of Quantics Consulting, Roslin BioCentre, Edinburgh, Scotland, UK.

## Authors’ contributions

SB: Served as principal investigator on the US study, evaluated the data at several times with ProStrakan, participated in forming the publication strategy, and provided review and approval of the final draft of the manuscript. CB: Performed clinical data acquisition, reviewed and commented on the intellectual content of the drafts, and gave approval to the final manuscript for publication. RB: Performed data collection and reviewed and approved the final draft of the manuscript. GC: Participated in face-to-face meetings with FDA to discuss study design, participated in development of the concept and design of the study, ensured data were collected accurately and in a timely manner in accordance with International Conference on Harmonisation (ICH) Guideline for Good Clinical Practice, ensured final data were reported in accordance with ICH guidelines and were suitable for submission in an NDA dossier, developed original draft of manuscript, and reviewed and approved the final draft of the manuscript. GVC: Served as study investigator, identified, recruited, and managed patients during the study, and reviewed and approved the final draft of the manuscript. JH: Participated in study design and conduct, provided analysis and interpretation of the data, and reviewed and approved the final draft of the manuscript. JP: Served as study investigator, identified, recruited, and managed patients during the study, and reviewed and approved the final draft of the manuscript. DR: Performed clinical data acquisition, served as study investigator, identified, recruited, and managed patients during the study, and reviewed and approved the final draft of the manuscript. MS: Participated in development of the original concept for the trial, participated in clinical recruitment, reviewed and revised the draft, and provided review and approval of the final draft of the manuscript. AY: Responsible for statistical aspects of study design, reviewed original statistical analysis and clinical study report, executed final statistical analysis, and reviewed and approved the final draft of the manuscript. All authors read and approved the final manuscript.

## Pre-publication history

The pre-publication history for this paper can be accessed here:

http://www.biomedcentral.com/1471-230X/13/106/prepub
